# Sexual communication of *Spodoptera frugiperda* from West Africa: Adaptation of an invasive species and implications for pest management

**DOI:** 10.1038/s41598-020-59708-7

**Published:** 2020-02-19

**Authors:** Sabine Haenniger, Georg Goergen, Mobolade Dele Akinbuluma, Maritta Kunert, David G. Heckel, Melanie Unbehend

**Affiliations:** 1 Department of Entomology, Max Planck Institute for Chemical Ecology, Hans-Knöll-Str. 8, 07745 Jena, Germany; 2 International Institute of Tropical Agriculture, 08 BP 0932 Tri Postal, Cotonou, Benin; 3 Department of Crop Protection and Environmental Biology, University of Ibadan, Ibadan, Nigeria; 4 Department of Natural Product Biosynthesis, Max Planck Institute for Chemical Ecology, Hans-Knöll-Str. 8, 07745 Jena, Germany

**Keywords:** Chemical biology, Ecology

## Abstract

The pest species *Spodoptera frugiperda*, which is native to North and South America, has invaded Africa in 2016. The species consists of two strains, the corn-strain and rice-strain, which differ in their sexual communication. When we investigated populations from Benin and Nigeria, consisting of corn-strain and rice-corn-hybrid descendants, we found no strain-specific sexual communication differences. Both genotypes exhibited the same pheromone composition, consisting of around 97% (Z)-9-tetradecenyl acetate (Z9–14:Ac), 2% (Z)-7-dodecenyl acetate (Z7–12:Ac), and 1% (Z)-9-dodecenyl acetate (Z9–12:Ac), they had similar electrophysiological responses, and all mated around three hours into scotophase. However, we found geographic variation between African and American populations. The sex pheromone of African corn-strain and hybrid descendant females was similar to American rice-strain females and showed higher percentages of the male-attracting minor component Z7–12:Ac. In addition, African males exhibited the highest antennal sensitivity towards Z7–12:Ac, while American males showed highest sensitivity towards the major pheromone component Z9–14:Ac. Increasing the production of and response to the critical minor component Z7–12:Ac may reduce communication interference with other African *Spodoptera* species that share the same major pheromone component. The implications of our results on pheromone-based pest management strategies are discussed.

## Introduction

Since 2016 the African continent has a new pest species: the fall armyworm (FAW) *Spodoptera frugiperda* (Lepidoptera: Noctuidae)^1^. This noctuid moth species is endemic to North and South America and is known as serious pest of maize, sorghum, sugarcane, and various grasses^2,3^. Although FAW is a generalist feeding on over 350 different host plants in the Americas^4^, its major host in Africa is maize^5^, also endemic to the Americas. The rapid spread of FAW to almost all sub-Saharan countries within less than two years^5^, and the magnitude of agricultural losses (estimated over US$ 13.3 billion^6^), highlight the need for efficient pest management strategies in Africa. As FAW encounters new ecological conditions, ecosystem dynamics, and species interactions in Africa, this invasive species may behave differently in its new environment, and pest control methods that work in the Americas might not be effective in Africa. Therefore, it is crucial to first understand the biology of FAW in its new habitat in order to develop control methods that are effective and affordable for regional farmers.

One commonly used and environmentally friendly method to monitor or control pest insects is the use of sex pheromones, which are crucial for the mate finding process in moths^7^. In most noctuid species like FAW, the females emit a species-specific sex pheromone in the scotophase to attract males over large distances, which is the start of the mating phase^8^. In its native environment in North and South America, several sex pheromone based pest management strategies have been used to regulate FAW populations, i.e. monitoring with pheromone traps to determine timing of pesticide application^9,10^, mass trapping to reduce population densities^11^, and mating disruption with high pheromone concentrations to disturb the mate finding process^12^. In Africa, monitoring with pheromone traps (baited with American lures) is currently used to assess infestation levels^13,14^ and preliminary field trials are performed to evaluate the efficiency of mating disruption^5^. The key component in these control methods is the sex pheromone of FAW females. Thus, it is essential to determine the pheromone composition of FAW females from Africa, which might be different compared to American populations.

The male attracting pheromone that FAW females from the Americas release consists of at least two components: the major component (Z)-9-tetradecenyl acetate (Z9–14:Ac) and the minor component (Z)-7-dodecenyl acetate (Z7–12:Ac)^15^. Two other minor compounds commonly found in the pheromone gland are (Z)-9-dodecenyl acetate (Z9–12:Ac) and (Z)-11-hexadecenyl acetate (Z11–16:Ac)^16,17^, but field tests in Florida did not show that they are important for male attraction^15,17^. The minor component (E)-7-dodecenyl acetate (E7–12:Ac) shows geographic variation and has so far only been found in females from Brazil^18^. With regard to pest management, it is important to consider that FAW consist of two strains (the corn-strain and the rice-strain) that exhibit differences in their pheromone composition^16,17^ and timing of reproduction in the night^19,20^. American corn-strain females, which mate early in the scotophase^19^, are known to produce smaller relative amounts of the critical male attracting minor component Z7–12:Ac compared to rice-strain females^16,17^, which are sexually active at the end of the scotophase^19^. A recent genetic study that determined the strain identity of African FAW populations has shown that the rice-strain population is rare or absent on the continent and mainly corn-strain individuals and descendants of interstrain hybrids are present^21^. However, it is so far unclear whether geographic variation exists between corn-strain populations from Africa and America, and whether there are corn-rice-strain hybrids in Africa which differ in their sexual behavior compared to pure strain individuals.

One factor that could influence the sexual communication system of FAW in Africa is the presence of communication interference with other species. Within Africa, 8 other species of the genus *Spodoptera* have been reported, i.e. *S. apertura*, *S. cilium*, *S. exempta*, *S. exigua*, *S. littoralis*, *S. mauritia*, *S. malagasy*, and *S. triturata*^22^. Interestingly, *S. cilium, S. exempta*, and *S. triturata* share the same major component Z9–14:Ac with FAW, in *S. exigua* and *S. littoralis* Z9–14:Ac has been reported as minor component, and the sex pheromone of *S. apertura*, *S. mauritia*, and *S. malagasy* is still unknown (http://www.pherobase.com). Thus, there is an overlap in the sex pheromone composition of FAW with at least five other *Spodoptera* species present in Africa. Besides other *Spodoptera* species, traps in Togo baited with FAW lures had high numbers of non-target catches of *Leucania loreyi*^13^, which also uses Z9–14:Ac as the major pheromone component and Z7–12:Ac as the minor component^23^. As FAW is now a broad generalist that occurs throughout Sub-Saharan Africa, there is a high probability that it encounters other species during its adulthood that share the same pheromone compounds, and communication interference may have caused a change in the sexual behavior of FAW in Africa compared to American populations. To test whether geographic and/or strain-specific variation exists in African FAW populations, we investigated the sexual communication system of FAW populations from West Africa (Benin and Nigeria) by using a combination of genetic, electrophysiological, chemical, and behavioral observation methods. The implications of our results on the adaptation of the invasive species to its new habitat and the development of regional pest management strategies are discussed.

## Results

### Pure corn-strain and RC hybrid descendants present in West Africa

Genetic strain-identification used both the maternally-inherited mitochondrial cytochrome oxidase I (COI) gene and the nuclear-encoded triose phosphate isomerase (TPI) gene. Offspring of interstrain hybrids are indicated when the strain-specific markers are discordant. For example, among the FAW population from Benin, 36% exhibited the C_COI_-C_TPI_ genotype (n = 32) and thus descent from a corn-strain female and a corn-strain male, while 64% had the R_COI_-C_TPI_ genotype (n = 56), suggesting descent from a rice-strain female and a corn-strain male. In Nigeria, 63% of the population consisted of the C_COI_-C_TPI_ genotype (n = 60) and 37% of the R_COI_-C_TPI_ genotype (n = 36). The TPI marker was homozygous C_TPI_ for all individuals from Benin and Nigeria that were sequenced. Thus, in both West African countries we only found pure corn-strain individuals (C_COI_-C_TPI_) and descendants of rice-corn interstrain hybrids (R_COI_-C_TPI_), but no pure rice-strain specimens (R_COI_-R_TPI_).

### Increased antennal sensitivity to the minor component Z7–12:Ac in African populations

**Figure 1:**
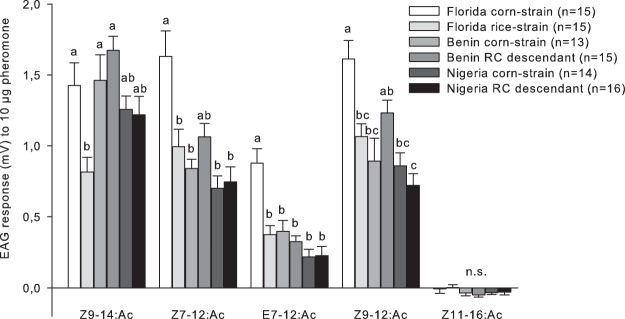
EAG responses of FAW males from Florida, Benin, and Nigeria to 10 µg of five different pheromone compounds. Bars show the mean pheromone response minus the hexane control response, with standard errors. Different letters above the bars indicate significant population differences for a given compound (ANOVA followed by Tukey’s HSD post hoc test; P < 0.05; n.s. = not significant).

In the electroantennogram (EAG) experiments we observed geographic and dose-response variation in the male response to five different compounds that were tested. At the lowest amounts of 0.001 µg and 0.1 µg pheromone, corn- and rice-strain males from Florida responded only to the major pheromone component Z9–14:Ac, while males from Benin only responded to the minor component Z7–12:Ac (Table 1). Males from Nigeria showed no significant EAG responses when exposed to 0.001 µg pheromone, but at 0.1 µg pheromone, only Z7–12:Ac evoked EAG responses that were significantly larger than the control (Table 1). At the highest amounts tested (10 µg), both American and African populations responded to four of five tested compounds, i.e. Z9–14:Ac, Z7–12:Ac, E7–12:Ac, and Z9–12:Ac evoked responses, but not Z11–16:Ac (Table 1, Fig. 1). The EAG responses to 10 µg pheromone revealed that for each of the five tested compounds, males of both genotypes from Benin or Nigeria exhibited similar EAG amplitudes, while the Florida corn-strain always showed higher EAG amplitudes than the Florida rice-strain (Fig. 1). For the major component Z9–14:Ac, African populations had similar EAG responses as the Florida corn-strain, while for all minor compounds the African populations were more similar to the Florida rice-strain (Fig. 1).

**Table 1:** Statistical analysis of FAW male EAG responses to three doses (0.001 µg, 0.1 µg and 10 µg) of five pheromone compounds (Z9–14:Ac, Z7–12:Ac, E7–12:Ac, Z9–12:Ac, Z11–16:Ac).

Region	Strain (genetic marker)	Z9–14:Ac	Z7–12:Ac	E7–12:Ac	Z9–12:Ac	Z11–16:Ac	Sample size
		Pheromone dose 0.001 µg^a^					
Florida	Corn-strain (C_COI_-C_TPI_)	**<0.001**	0.247	1.000	1.000	0.984	n = 15
	Rice-strain (R_COI_-R_TPI_)	**<0.001**	0.925	1.000	1.000	1.000	n = 15
Benin	Corn-strain (C_COI_-C_TPI_)	0.895	**0.034**	0.166	0.985	0.462	n = 13
	RC descendant (R_COI_-C_TPI_)	1.000	**<0.001**	0.999	0.752	0.991	n = 15
Nigeria	Corn-strain (C_COI_-C_TPI_)	0.991	1.000	1.000	0.996	1.000	n = 14
	RC descendant (R_COI_-C_TPI_)	1.000	0.549	1.000	1.000	1.000	n = 16
		**Pheromone dose 0.1 µg**^**a**^					
Florida	Corn-strain (C_COI_-C_TPI_)	**<0.001**	0.206	1.000	0.402	0.978	n = 15
	Rice-strain (R_COI_-R_TPI_)	**<0.001**	0.988	0.998	0.477	1.000	n = 15
Benin	Corn-strain (C_COI_-C_TPI_)	1.000	**<0.001**	1.000	1.000	0.996	n = 13
	RC descendant (R_COI_-C_TPI_)	0.980	**<0.001**	0.879	1.000	1.000	n = 15
Nigeria	Corn-strain (C_COI_-C_TPI_)	1.000	**0.001**	1.000	1.000	0.998	n = 14
	RC descendant (R_COI_-C_TPI_)	1.000	**0.015**	0.993	0.999	1.000	n = 16
		**Pheromone dose 10 µg**^**a**^					
Florida	Corn-strain (C_COI_-C_TPI_)	**<0.001**	**<0.001**	**<0.001**	**<0.001**	1.000	n = 15
	Rice-strain (R_COI_-R_TPI_)	**<0.001**	**<0.001**	**<0.001**	**<0.001**	1.000	n = 15
Benin	Corn-strain (C_COI_-C_TPI_)	**<0.001**	**<0.001**	**<0.001**	**<0.001**	1.000	n = 13
	RC descendant (R_COI_-C_TPI_)	**<0.001**	**<0.001**	**<0.001**	**<0.001**	0.979	n = 15
Nigeria	Corn-strain (C_COI_-C_TPI_)	**<0.001**	**<0.001**	**0.023**	**<0.001**	1.000	n = 14
	RC descendant (R_COI_-C_TPI_)	**<0.001**	**<0.001**	**0.040**	**<0.001**	1.000	n = 16

^a^Shown are P-values (ANOVA followed by a Tukey’s HSD post hoc test), where the EAG pheromone response was compared to the control (hexane) response. Bold fields highlight the components that were significantly different to the control stimuli (P < 0.05).

### No strain-specific pheromone variation in different African genotypes

Gas chromatography-mass spectrometry (GC-MS) analysis showed that corn-strain and RC hybrid descendant females from Benin and Nigeria produced four out of five analyzed pheromone compounds that have been previously found in American populations, i.e. Z9–14:Ac, Z7–12:Ac, Z9–12:Ac, and Z11–16:Ac are present, but not E7–12:Ac (Supplementary Fig. S1).

**Figure 2:**
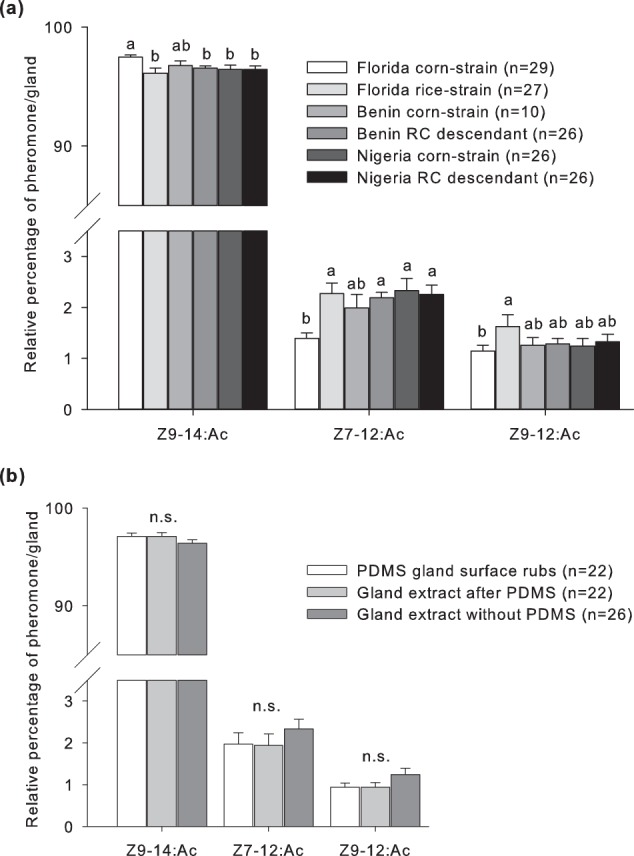
Pheromone gland composition of FAW females from Florida, Benin, and Nigeria. (**a**) Mean relative percentages of Z9–14:Ac, Z7–12:Ac, and Z9–12:Ac, with standard errors, of hexane gland extracts. Data from field populations from Florida have been previously published^17^ and were included for comparison. (**b**) Pheromone composition of Nigeria corn-strain females using three different pheromone extraction methods (gland surface rubs with polydimethylsiloxane (PDMS) fibers, hexane gland extracts after PDMS rubs, and hexane gland extracts without previous PDMS rubs). Different letters above the bars indicate significant differences for a given compound (GLM analysis; P < 0.05, n.s. = not significant).

Of the four pheromone compounds that have been identified in the glands of African females (Supplementary Fig. S1), EAG experiments revealed that males were not responsive to Z11–16:Ac (Table 1), which is why we focused in the individual gland analysis on the three components that are present in the gland and evoked EAG responses: the major component Z9–14:Ac, and the two minor compounds Z7–12:Ac and Z9–12:Ac. Analyses of hexane extracts of the pheromone glands of African females revealed that corn-strain and RC hybrid descendants from Benin and Nigeria had the same pheromone profile (Fig. 2a). On average, African females exhibited 96.4–96.8% Z9–14:Ac, 2.0–2.3% Z7–12:Ac, and 1.2–1.3% Z9–12:Ac (Fig. 2a). In comparison with the American populations, the African females had similar percentages of Z9–14:Ac and Z7–12:Ac as the Florida rice-strain, while the relative amount of Z9–12:Ac was not significantly different between African and American populations (Fig. 2a). The total amount of pheromone in single FAW glands ranged between 8.5 up to 46.6 ng (Supplementary Table S1).

To prove that the relative pheromone amount in the gland is similar to the pheromone released to the gland surface, we compared hexane gland extracts with gland surface extracts using fused silica optical fibers coated with polydimethylsiloxane (PDMS)^24^ and discovered that the percentages of Z9–14:Ac, Z7–12Ac, and Z9–12:Ac were not significantly different between both methods (Fig. 2b). However, when using the PDMS technique, the relative amount of Z7–12:Ac (~2%) and Z9–12:Ac (~0.9%) was on average smaller compared to the hexane gland extraction method without previous PDMS treatment (Z7–12:Ac up to 2.3%, Z9–12:Ac up to 1.3%, Fig. 2b). With the PDMS gland surface rubs, we were able to detect around 23% of the total pheromone collected with the hexane gland extraction method (~10.6 ng with PDMS rubs vs. ~46.6 ng with gland extracts after PDMS rubs, Supplementary Table S1).

### Early mating times of African populations

**Figure 3:**
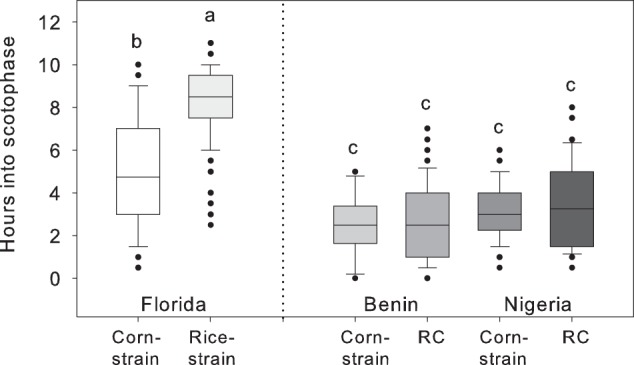
Mating times of FAW populations from Florida, Benin, and Nigeria. Boxplots show the onset time of first mating of two populations from Florida (corn-strain: n = 194, rice-strain: n = 189) and four populations from Africa (Benin corn-strain n = 9, Benin RC hybrid descendant: n = 82, Nigeria corn-strain: n = 64, Nigeria RC hybrid descendant: n = 38). Data from laboratory populations from Florida have been previously published^20^ and were included for comparison. Different letters above the bars indicate significant differences (ANOVA followed by Tukey’s HSD post hoc test; P < 0.05).

Observation of the mating behavior in the laboratory showed that all four African populations mated early, on average around three hours in the scotophase (Fig. 3). In the American populations, the Florida corn-strain mated around five hours into scotophase, and the Florida rice-strain on average eight hours into scotophase (Fig. 3).

## Discussion

When we investigated the sexual communication system of FAW populations from West Africa, consisting of corn-strain individuals (C_COI_-C_TPI_) and descendants of interstrain hybrids (R_COI_-C_TPI_), we found no phenotypic differences between the two genotypes, i.e. they exhibited the same EAG responses, pheromone profiles, and onset time of mating. Of these three measured phenotypes, the best defined strain-specific difference and proposed prezygotic mating barrier of FAW populations from America is their onset time of mating, with the corn-strain mating approximately 3 hours earlier than the rice-strain^19,25^. Behavioral observations of F1 hybrid individuals from America showed that crosses between a rice-strain female and a corn-strain male (R_COI_-C_TPI_) exhibit similar mating times as the pure rice-strain, i.e. they mate at the end of the scotophase^19^. In our study, the African populations with the R_COI_-C_TPI_ genotype were sexually active at the beginning of the scotophase, even two hours earlier than the corn-strain individuals from Florida (Fig. 3). Along with the fact that the TPI marker was not heterozygous, this suggests that individuals with R_COI_-C_TPI_ genotype from Africa are not F1 hybrids but must be F2 or later generations after the initial hybridization. The early mating time of the African populations indicates that the genetic basis of allochronic differentiation, suggested to be the circadian clock gene *vrille* on chromosome 25 and possibly other clock genes identified by a previous study^20^, was inherited from the corn-strain in both genotypes (C_COI_-C_TPI_ and R_COI_-C_TPI_). Interestingly, genetic analyses of the pheromone production of FAW females identified the same chromosome 25, on which *vrille* is located, to be involved the production of the critical minor pheromone component Z7–12:Ac^26^. For both African genotypes (C_COI_-C_TPI_ and R_COI_-C_TPI_) we found similar relative percentages of Z7–12:Ac as reported for American rice-strain females^16,27^, which suggests that the gene(s) on FAW chromosome 25 responsible for Z7–12:Ac production were inherited from the rice-strain in the African populations. Now the question arises of whether the African corn-strain is really a pure corn-strain as found in America, or if repeated matings between corn-strain individuals with rice-corn-strain hybrid descendants substantially changed the African corn-strain genome. Currently, only two genes are routinely used to genetically analyze FAW populations: the TPI gene on the sex chromosome^28^ and the COI gene on the mitochondrial genome^29^. Without more reliable strain-specific autosomal markers or whole genome sequence comparisons, it is difficult to estimate how much of the genome from the corn-strain and R_COI_-C_TPI_ hybrid descendants is inherited from the corn- or the rice-strain, and how a mixed genetic basis might influence certain behaviors in the field. The genome sequences of both strains were published in 2017, and comparative analyses between the strains identified significant variation in a number of detoxification and digestions genes, which might be linked to differential host plant use^30^. These findings could serve as starting point to establish new screening markers for FAW genotyping or be the basis for genome comparisons in order to determine the genetic architecture of FAW populations in Africa.

A comparison between the sexual communication system of populations from America and Africa revealed two (possibly linked) differences. First, we found an increased relative amount of the critical male attracting minor component Z7–12:Ac in African corn-strain and RC hybrid descendant females, similar to American rice-strain females (Fig. 2). Second, males from African populations revealed the highest pheromone sensitivity towards Z7–12:Ac in the EAG experiments, while American males exhibited the highest sensitivity towards the major component Z9–14:Ac (Table 1). Increasing the production of and response to the critical minor component Z7–12:Ac may reduce communication interference with other African *Spodoptera* species that share the same major pheromone component, but whether this has resulted from sexual selection in FAW in the new environment requires future work. The elevated levels of Z7–12:Ac in African FAW females could be the result of rice-strain specific pheromone production genes, which may have been inherited to the present C_COI_-C_TPI_ and R_COI_-C_TPI_ genotypes by multiple inter-strain specific crosses within the initial invasive population. Another explanation could be that FAW females exhibit phenotypic plasticity in their sexual communication, a phenomenon reported for the noctuid moth *Heliothis subflexa*^31^. Laboratory *H. subflexa* females, which were exposed for the first days of their adult lives to the sex pheromone of the closely related *H. virescens*, changed their pheromone composition by producing significantly more Z11–16:Ac, a compound inhibiting attraction of *H. virescens* males^31^. Early-adult experience of heterospecific pheromone blends could also affect the pheromone composition of FAW females, for example by influencing transcription, translation, or activity levels of so far uncharacterized enzymes involved in Z7–12:Ac production. With regard to the increased antennal sensitivity of African males towards Z7–12:Ac, which was not present in American males (Table 1), adaptive phenotypic plasticity may also play a role. Heterospecific pheromone blends could alter, for example, the expression levels of Z7–12:Ac receptors on the male antennae, or change the abundance of transporting proteins like odorant-binding proteins or chemosensory proteins, which are known to deliver semiochemicals to receptor neurons and thereby contributing to the sensitivity and selectivity of the insect’s olfactory system^32,33^. Understanding the reasons for the sexual communication differences between the native and invasive FAW populations will give us a deeper insight into the adaptation and evolution of invasive species in new environments. Also, the increased male antennal sensitivity to Z7–12:Ac in African populations harbors the possibility to enhance the efficiency of mating disruption with specific pheromone dispensers in Africa. This should be explored in field studies in the near future.

When we observed the mating times of FAW pairs, all African populations mated on average three hours in the scotophase (Fig. 3), which is two hours earlier than previously reported for the corn-strain from America^19^. This time shift could be an additional mechanism to avoid cross attraction with other species. *Spodoptera exempta*, which shares the same major component with FAW^34^, has been reported to mate between 6–9.5 hours into scotophase^35^. This mating phase overlaps with the mating time of corn- and rice-strain FAW populations from Florida^19^, so that advancing the reproductive phase by two hours could be advantageous for FAW populations in Africa. However, the time shift between the Florida corn-strain and the African populations could also have developed by genetic drift during laboratory rearing, as the previous study used insects that were reared for ~6 years in the laboratory^20^, while we used populations kept in the laboratory for 4 months or less. This will be tested in the future by observing fresh field populations from Florida. While 4 months of laboratory rearing might not have contributed to genetic changes that could have an influence on the results, it is worthwhile mentioning that laboratory conditions are lacking parameters of field conditions, like plant odors, plant diet, predators, and parasitoids, which could influence the tested phenotypes. It is also important to mention that we observed the mating behavior under a 14:10 L:D cycle, in order to compare it with the previous studies using American populations^19,20^, whereby the natural L:D cycle is 12:12 in Benin and Nigeria. Shortening the night cycle of the African populations by two hours could have also contributed to the observed two hours mating time shift. Additional observation experiments of field populations from America and other regions in Africa, with different natural L:D cycles, will prove whether the mating time of FAW can be influenced by laboratory rearing and/or the L:D cycle.

With regard to the development of a pheromone based pest management system for FAW in Benin and Nigeria (with C_COI_-C_TPI_ and R_COI_-C_TPI_ genotypes), genotype-specific differences may not need to be considered when targeting the invasive species with pheromones. Our findings that the FAW populations from Benin and Nigeria consist of corn-strain individuals and descendants of interstrain hybrids, but not of pure rice-strain individuals, are in accordance with previous genetic analyses^21^. As we sampled only a small proportion of both field populations (Benin n = 88 individuals and Nigeria n = 96 individuals), we cannot exclude that rice-strain specimens might be present in these regions and genotype specific sexual communication differences may exist in Benin, Nigeria or other African countries. In general, American lures have been successfully used to catch FAW males in Africa, although high numbers of non-target moths, some of which have similar appearance as FAW and might not be distinguished by local farmers^13^, highlight the need for more specific pheromone formulations. Analysis of the composition of American lures showed that lures like “L105A” from Scentry Biologicals, Inc. (Billings, Montana), the “FAW” lure from Trécé, Inc. (Adair, Oklahoma), and the “Scenturion Fall Armyworm Lure” from Suterra LLC (Bend, Oregon) contain Z11–16:Ac^36^, a compound not detected by the male antennae in EAG experiments with populations from America^18,37^ and Africa (Table 1). Thus, this compound is most likely not needed for male attraction, but its addition to FAW lures might attract other species. Dose response experiments with the important minor component Z7–12:Ac, which is produced by females in concentrations of less than 5% (Fig. 2), showed a decrease in male attraction when high percentages of 5–10% Z7–12:Ac were added to the lures^11,17^. The fact that some American lures contain extremely high percentages of Z7–12:Ac, i.e. the “FAW” lure from Trécé releases almost 30% Z7–12:Ac, the “L105A” and “L976” lures from Scentry Biologicals around 10% Z7–12:Ac, and the “Scenturion Fall Armyworm Lure” circa 8% Z7–12:Ac^13^, could explain the attraction of non-target moths like *Leucania loreyi* to traps baited with FAW lures^13^. The results of our study showed that Z9–14:Ac, Z7–12:Ac, and Z9–12:Ac are the most promising candidates that should be used to formulate FAW lures, as only these three compound are present within the female gland and evoked EAG responses larger than the control stimuli (Figs. 1 and 2). Based on the relative pheromone percentages that we found in the female gland (Fig. 2), lures consisting of 2% Z7–12:Ac + 98% Z9–14:Ac should be compared with lures containing 1% Z9–12:Ac + 2% Z7–12:Ac + 97% Z9–14:Ac, in order to determine whether Z9–12:Ac is a male attracting component, which is not the case in America^17^. Additionally, the total pheromone amounts should be taken into account, ranging in our experiments between 8.5–46.6 ng of pheromone per female gland (Supplementary Table S1). Dose response experiments in Brazil showed that when testing 10 mg, 1 mg, 0.1 mg, and two females as the pheromone source, more males were caught in traps baited with 1 mg pheromone^18^. Whether these concentrations will work as effectively in Africa as in America depends also in the release rate of the lure itself and still needs to be investigated. In summary, we are just beginning to understand the biology of FAW in its new environment and the more insight we will get into its adaptation mechanisms, the better we can manage the spread of this agricultural pest species.

## Methods

### Insects

The FAW populations from Nigeria originated from ~270 larvae collected from corn fields in Ijaiye Farm Settlements, in Akinyele local government area of Oyo State (7°39′07.6″N; 3°49′51.2″E) in 2018. The field larvae from Nigeria were shipped to Germany in October 2018. Populations from Benin originated from mass rearing colonies at IITA Cotonou. This population was established from larvae collected in corn fields in 2016 and mated every second month to field individuals collected from corn plants. In December 2018, egg masses from Benin were shipped to Germany where ~600 larvae emerged. The Florida rice-strain was collected in a grass field in Moore Haven (+26° 53′ 3.04′′, −81° 7′ 21.17′′) in May 2010 (~300 larval specimens), and the corn-strain originated from corn plots at a University of Florida research station in Citra, northern Marion county, in September 2018 (~200 larval specimens).

All specimens (from Florida, Nigeria and Benin) were shipped to Germany, where they were reared on artificial pinto bean diet in climate chambers at 26 °C and 70% RH, with reversed light:dark (L:D) cycle and 14:10 L:D photoperiod. Adults were fed with 10% honey water and mated randomly in single pairs. All adult pairs from the field that produced fertile offspring were genetically analyzed to identify their strain (see below) and genotype-specific colonies were established and continuously reared. All experiments were conducted in Germany. Experiments with African populations and the Florida corn-strain were conducted within the first 4 months of laboratory rearing, while the Florida rice-strain was reared for eight years in the laboratory in Germany before experiments were conducted. Unfortunately, we were not able to maintain a stable corn-strain population from Benin (C_COI_-C_TPI_) in the laboratory, which is why experiments conducted with this population usually have lower sample sizes than the other populations.

### Genetic strain identification

Strain identification was assessed by screening adults for one mitochondrial marker (COI) and one nuclear marker (TPI), which are known to be diagnostic for both strains in North and South America^28,29^. DNA extractions were performed in a 96-well plate using Chelex 100 Resin (Bio-Rad Laboratories, Hercules, CA, USA). One adult leg was put in one well together with two metal beads and 300 µl 10% Chelex (diluted in ddH_2_O). The tissue was homogenized in a tissue lyser for 4 min at 30 Hz. The samples were heated for 30 min at 95 °C and 300 rpm spinning, after which they were frozen at −20 °C overnight. Then each plate was thawed, mixed, and centrifuged at 4000 rpm for 30 min. The supernatant was filtered through a fritted deep well filter plate (Thermo Fisher Scientific, Waltham, MA, USA) and used for strain analyses. Identification of the mitochondrial marker was performed as described by Unbehend, *et al*.^17^, i.e. after amplification of the COI gene, two strain-specific digests with MspI and SacI were conducted to analyze the strain-affiliation via gel electrophoresis. The TPI marker was analyzed according to methods used by Nagoshi^28^, i.e. a part of the TPI gene was Sanger-sequenced at the MPI-CE and ten single nucleotide polymorphisms were used for strain identification. The sequences were analyzed with Sequencher 5.2.4 (Gene Codes Corporation, Ann Arbor, MI, USA).

### Electrophysiology

To identify the male antennal response to Z9–14:Ac, Z7–12:Ac, E7–12:Ac, Z9–12:Ac, and Z11–16:Ac, EAG recordings of 0–5 days old virgin males were performed with populations from Benin, Nigeria, and Florida. Samples from Florida were included in the analysis as we found no study in the literature that analyzed the EAG response of American populations to all five compounds. Male heads were severed, gently crushed to eliminate movement of the antennae, and placed on a fork-shaped electrode holder (Syntech, Buchenbach, Germany) using an electrolyte gel (Spectra 360 Electrode Gel, Parker Laboratories, Fairfield, NJ, USA). Three amounts (0.001 µg, 0.1 µg, and 10 µg) of each pheromone compound were presented from the lowest to the highest concentration. The lowest pheromone amount (0.001 µg) and 100 times higher amount of 0.1 µg pheromone were chosen as they reflect the range of the female pheromone production. The highest amount of 10 µg pheromone was used as maximum stimulus to identify responses that might not be detected at lower amounts. Ten µl of each pheromone component in each amount were pipetted onto a small piece of filter paper (1 cm^2^) and placed into a glass Pasteur pipette. Control pipettes contained filter paper with 10 µl hexane alone. A stimulus controller (CS-55, Syntech) produced a flow of 1 l/min charcoal-filtered air over the antennae. Antennae were stimulated with 0.5 s odor puffs, using a randomized sequence for each recording. Before and after testing three different odors, the control stimulus was presented. The inter-stimulus interval was 30 s. Signals were recorded with Syntech software (Autospike Version 3.9) and statistically analyzed with a one-way analysis of variance (ANOVA) followed by a Tukey’s HSD post hoc test in R version 3.2.0^38^. To meet assumptions of normality and variance homogeneity, the spike amplitude (in mV) was log(x + 1) transformed.

### Pheromone analysis

The sex pheromone of 2–4 days old virgin females from Benin and Nigeria was analyzed in three experiments. Pheromone data of FAW females from Florida have been previously described by Unbehend, *et al*.^17^ and were included in this manuscript for comparison. In the first experiment, GC-MS analysis was performed to evaluate whether the five pheromone compounds that have been found in FAW females from America (Z9–14:Ac, Z7–12:Ac, E7–12:Ac, Z9–12:Ac, Z11–16:Ac) are also present in populations from Africa. Glands were extracted 2–4 h into scotophase. The gland was excised from the female abdomen and singly placed into a glass vial containing 50 µl hexane (Carl Roth, Karlsruhe, Germany) plus 125 ng pentadecane (Sigma-Aldrich Chemie GmbH, Munich, Germany) as internal standard. After 30 min, the gland was removed and extracts of 10 females per region and strain were pooled and reduced from 500 µl to 30–50 µl under a gentle stream of nitrogen. Three µl of the reduced extract were analyzed with GC-MS (5977 A MSD and 7890B GC Systems, Agilent technologies Inc., Santa Clara, CA, USA). The GC was equipped with an INNOWAX column (30 m, 0.25 mm id, 25 μm film thickness, Agilent Technologies) and helium was the carrier gas (1 ml/min). The inlet temperature was 240 °C. The GC was programmed from 60 °C with a 2 min hold to 180 °C at 30 °C/min, then to 230 °C at 5 °C/min, and finally to 260 °C at 20 °C/min with a final 5 min hold. The MS transfer line was held at 260 °C, the MS ion source at 230 °C, and mass spectra were taken in EI mode (at 70 eV) in the range from 29–350 m/z. An internal standard containing 100 ng Z9–14:Ac, Z7–12:Ac, E7–12:Ac, Z9–12:Ac, and Z11–16:Ac (Pherobank, Wageningen, The Netherlands) was used as reference to confirm the presence of compounds by comparing retention times and mass spectra. To verify the presence of the isomers Z7–12:Ac and E7–12:Ac, selected ion monitoring (SIM) was conducted for the masses 67 and 81 with a dwell time of 50 ms.

In the second experiment, we extracted glands of single females from Benin and Nigeria and determined the relative and total amount of pheromone by GC analysis. Glands were extracted as described above and GC analysis was conducted on a HP7890 GC with a splitless inlet attached to a high resolution polar capillary column (DB-WAXetr (extended temperature range)) and a flame-ionization detector (FID). Chemical analysis and GC programming was performed as described by Unbehend, *et al*.^17^. Gland extracts were concentrated from the volume of 50 µl to 4 µl, after which the whole extracts were injected into the GC. To confirm retention times, an internal standard containing 100 ng Z9–14:Ac, Z7–12:Ac, E7–12:Ac, Z9–12:Ac, and Z11–16:Ac, and pentadecane was injected into the GC. All pheromone data were log transformed to stabilize the variance and analyzed using a generalized linear model (GLM) in R^38^.

In the third experiment, we assessed whether the pheromone composition in the gland was similar to the pheromone release from the gland and sampled pheromones from the gland surface of calling females with fused silica optical fibers coated with PDMS.

The PDMS sampling was conducted with one population, the corn-strain from Nigeria, according to methods described by Lievers and Groot^24^. PDMS fibers (100 μm polydimethylsiloxane; Polymicro Technologies Inc., Phoenix, AZ, USA) were cut in 15 mm long pieces and rubbed for 2.5 min over the pheromone gland of females that called for at least for 15 min, after which the fibers were extracted for 30 min in 50 µl hexane and 125 ng pentadecane. After PDMS sampling, the pheromone glands of the already sampled females were excised from the female abdomen and extracted as described above. All extracts were analyzed via GC as described above.

### Mating observations

To determine the onset time of mating, single pair matings of African populations were observed as described by Schöfl, *et al*.^19^. One to four day-old virgin adults were set up in single pairs in clear plastic cups (16 oz.) and fed with 10% honey solution. Matings were set up during the photophase and placed in a walk-in climate chamber (26 °C, 70% RH, L:D 14:10) at least two hours before scotophase. Couples were observed throughout the 10 hours of scotophase, with a 30 min interval. All pairs were observed for one or two consecutive nights starting at the first night after the mating. The onset time of the first mating, regardless of whether it occurred in the first or second night, was used for comparing the timing of mating behavior. Mating behavior of laboratory populations from Florida were previously described by Haenniger, *et al*.^20^ and included for comparison. Data were log transformed and analyzed with an ANOVA followed by a Tukey’s HSD post hoc test in R^38^.

## Supplementary information

Supplementary Information.

## Data Availability

All generated and analyzed data in this study are included in the main text or are available from the Edmond repository https://dx.doi.org/10.17617/3.3c.
