# Supplementary material for: Sexual communication of Spodoptera frugiperda from West Africa: Adaptation of an invasive species and implications for pest management
Source: Sci Rep. 2020 Feb 19;10:2892. doi: 10.1038/s41598-020-59708-7 (PMC7031376; doi:10.1038/s41598-020-59708-7)
Supplement: Supplementary file 1 — Supplementary Information. [file 41598_2020_59708_MOESM1_ESM.pdf]

Supplementary Information

Sexual communication of *Spodoptera frugiperda* from West Africa: Adaptation of an invasive species and implications for pest management

Sabine Haenniger[1], Georg Goergen[2], Mobolade Dele Akinbuluma[3], Maritta Kunert[4], David G. Heckel[1], Melanie Unbehend[1*]

[1]Department of Entomology, Max Planck Institute for Chemical Ecology, Hans-Knöll-Str. 8, 07745 Jena, Germany

[2]International Institute of Tropical Agriculture, 08 BP 0932 Tri Postal, Cotonou, Benin

[3]Department of Crop Protection and Environmental Biology, University of Ibadan, Ibadan, Nigeria

[4]Department of Natural Product Biosynthesis, Max Planck Institute for Chemical Ecology, Hans-Knöll-Str. 8, 07745 Jena, Germany

*Corresponding author. Tel.: +49 (0) 3641 57 1565, munbehend@ice.mpg.de

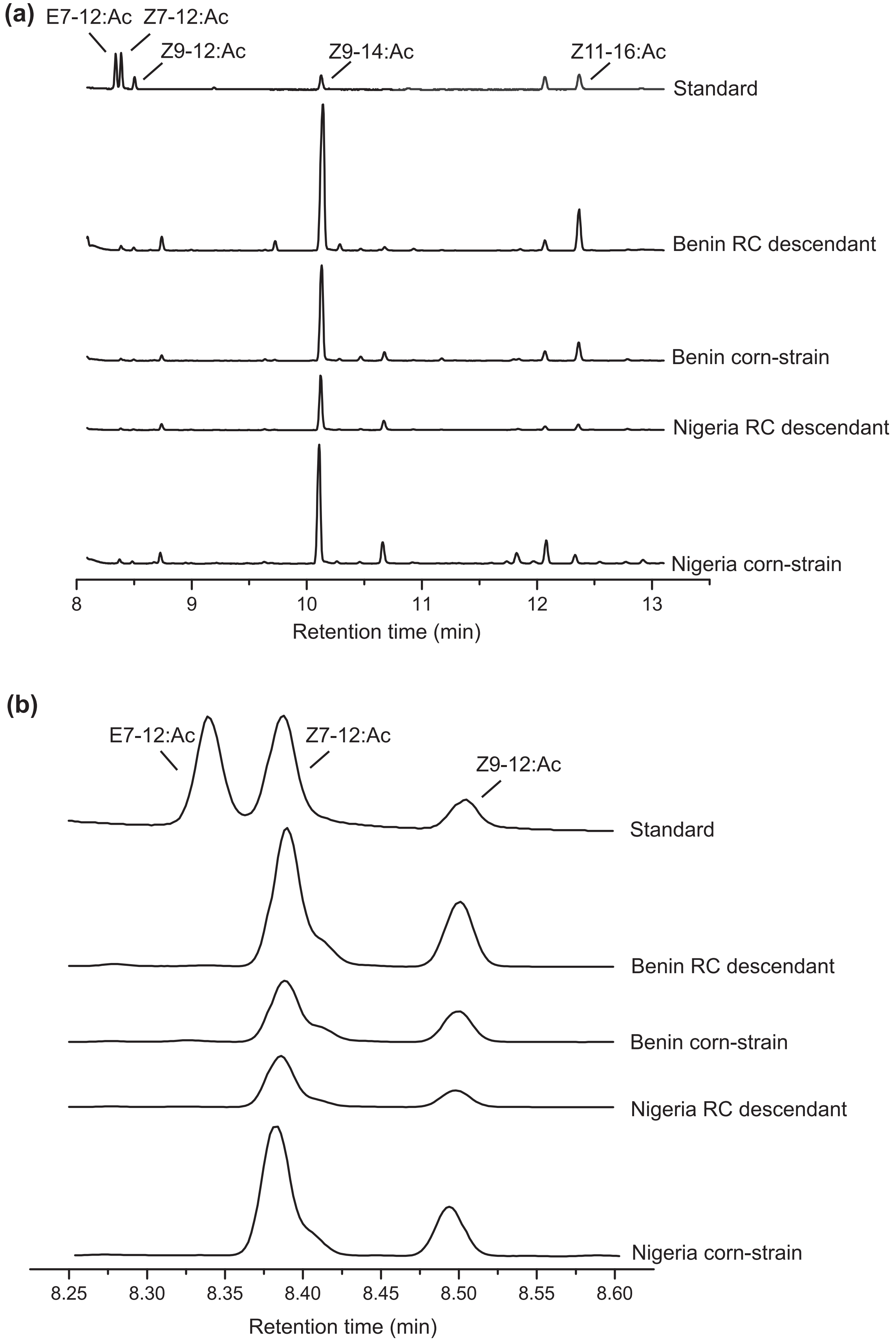

Supplementary Figure S1: Gas-chromatographic analysis of FAW pheromone glands from Benin and Nigeria. (a) Pheromone profiles of African FAW females compared with an standard containing five different compounds (E7-12:Ac, Z7-12:Ac, Z9-12:Ac, Z9-14:Ac, Z11-16:Ac). Each chromatogram represents a pooled sample of ten female pheromone glands. (b) Chromatogram of the selected ion monitoring run to confirm the absence of E7-12:Ac in African populations.

Supplementary Table S1. Total pheromone amount of FAW females from Florida, Benin, and Nigeria.

Region	Strain (genetic marker)	Extraction	Total amount in ng[2]	Sample size
Florida[1]	Corn-strain (C_{COI}-C_{TPI})	gland	11.5 ± 1.8[c]	n=29
	Rice-strain (R_{COI}-R_{TPI})	gland	13.8 ± 1.7[c]	n=27
Benin	Corn-strain (C_{COI}-C_{TPI})	gland	20.0 ± 8.3[bc]	n=10
	RC descendant (R_{COI}-C_{TPI})	gland	8.5 ± 1.3[c]	n=26
Nigeria	Corn-strain (C_{COI}-C_{TPI})	gland	35.3 ± 9.6[ab]	n=26
	RC descendant (R_{COI}-C_{TPI})	gland	25.7 ± 5.2[ab]	n=26
Nigeria	Corn-strain (C_{COI}-C_{TPI})	PDMS	10.6 ± 3.9[c]	n=22
		gland after PDMS	46.6 ± 11.5[a]	n=22

[1]Data from field populations from Florida have been previously published[16] and were included for comparison.
[2]Shown are mean values ± standard errors. Different superscript letters indicate significant differences (GLM analysis; $P<0.05$).
